# Identifying and preventing fraudulent responses in online public health surveys: Lessons learned during the COVID-19 pandemic

**DOI:** 10.1371/journal.pgph.0001452

**Published:** 2023-08-23

**Authors:** June Wang, Gabriela Calderon, Erin R. Hager, Lorece V. Edwards, Andrea A. Berry, Yisi Liu, Janny Dinh, August C. Summers, Katherine A. Connor, Megan E. Collins, Laura Prichett, Beth R. Marshall, Sara B. Johnson

**Affiliations:** 1 Johns Hopkins University Krieger School of Arts and Sciences, Baltimore, Maryland, United States of America; 2 Department of Pediatrics, Johns Hopkins School of Medicine, Baltimore, Maryland, United States of America; 3 Department of Population, Family and Reproductive Health, Johns Hopkins Bloomberg School of Public Health, Baltimore, Maryland, United States of America; 4 School of Community Health and Policy, Morgan State University, Baltimore, Maryland, United States of America; 5 Department of Pediatrics, University of Maryland School of Medicine, Baltimore, Maryland, United States of America; 6 Johns Hopkins Center for Communications Programs, Baltimore, Maryland, United States of America; 7 Johns Hopkins Wilmer Eye Institute, Baltimore, Maryland, United States of America; Kasturba Medical College, Mangalore, Manipal Academy of Higher Education, INDIA

## Abstract

Web-based survey data collection has become increasingly popular, and limitations on in-person data collection during the COVID-19 pandemic have fueled this growth. However, the anonymity of the online environment increases the risk of fraudulent responses provided by bots or those who complete surveys to receive incentives, a major risk to data integrity. As part of a study of COVID-19 and the return to in-person school, we implemented a web-based survey of parents in Maryland between December 2021 and July 2022. Recruitment relied, in part, on social media advertisements. Despite implementing many existing best practices, we found the survey challenged by sophisticated fraudsters. In response, we iteratively improved survey security. In this paper, we describe efforts to identify and prevent fraudulent online survey responses. Informed by this experience, we provide specific, actionable recommendations for identifying and preventing online survey fraud in future research. Some strategies can be deployed within the data collection platform such as careful crafting of survey links, Internet Protocol address logging to identify duplicate responses, and comparison of client-side and server-side time stamps to identify responses that may have been completed by respondents outside of the survey’s target geography. Other strategies can be implemented during the survey design phase. These approaches include the use of a 2-stage design in which respondents must be eligible on a preliminary screener before receiving a personalized link. Other design-based strategies include within-survey and cross-survey validation questions, the addition of “speed bump” questions to thwart careless or computerized responders, and the use of optional open-ended survey questions to identify fraudsters. We describe best practices for ongoing monitoring and post-completion survey data review and verification, including algorithms to expedite some aspects of data review and quality assurance. Such strategies are increasingly critical to safeguarding survey-based public health research.

## Background

Web-based survey data collection has become increasingly popular, and limitations on in-person data collection during the COVID-19 pandemic have fueled this growth [1–3]. Internet survey software and data capture systems (e.g., REDCap [4], Qualtrics (Qualtrics, Provo, UT)) can reduce effort and expenditures associated with recruiting participants and may, in some applications, assist with accessing populations who may be difficult to reach via other means [5–7]. Furthermore, the relative anonymity provided by online surveys may facilitate research involving marginalized communities or when respondents may otherwise be hesitant to disclose sensitive information [8–10].

Despite potential benefits related to access, online survey research (particularly if offering incentives for completion) presents an increased risk of fraudulent activity as compared to face-to-face data collection. There is evidence that fraud in research surveys has challenged past studies across public health disciplines, though few studies report on their techniques for detecting invalid data, if any such techniques have been implemented [1, 11]. As such, the extent to which fraud has impacted online research may not yet be entirely understood. While incentives can promote higher survey response and completion, they are also accompanied by an increased risk of interference from fraudulent responses [6, 12].

“Fraudsters” have various methods for finding surveys that involve incentives; for example, Meta (Facebook’s parent company) has an Ads Library that can help fraudsters find incentivized surveys that are advertised on their social media platforms, such as Facebook and Instagram. This resource can be exploited by fraudsters who may not be the intended target of a survey but may complete the survey solely for the incentive (“professional survey takers”) or utilize computer code (this technique is often referred to as “bots” or “botting”) to rapidly automate the completion of multiple surveys to receive multiple incentives.

Fraudsters pose a significant threat to research studies by undermining data quality and wasting resources [2, 10, 13]. Past recommendations for mitigating online survey fraud have included adding survey questions that reduce careless or automatic responding, implementing separate eligibility screeners, and implementing a Computer Automated Public Turing Test to tell Computers and Humans Apart (CAPTCHA) to outwit computer code that creates automatic responses [13]. However, as fraudsters continue to fine-tune their tactics, existing recommendations may not adequately distinguish between legitimate and fraudulent respondents. Additionally, fraudulent respondents may attempt to outwit preliminary eligibility screeners by repeatedly testing combinations of responses to identify the combination that meets inclusion criteria and thereby grants them access to the survey (known in cybersecurity as a “brute force” approach). Once identified, fraudsters may complete multiple surveys using the same criteria. Some fraudsters may use computer software that automatically completes surveys in rapid succession, producing many fraudulent responses (often referred to as “bot responses”). This can result in a disproportionate number of responses with identical or near-identical responses and poses a significant threat to validity should such tampering remain unidentified and unmitigated.

In this paper, we describe specific strategies and lessons learned from efforts to identify and address fraudulent online survey responses as part of a mixed-methods study of parents’ perceptions of public health recommendations related to COVID-19 outbreak mitigation in schools.

## Methods

The Parents and Communities as Experts (PACE) Study sought to understand how parents of students in kindergarten through grade eight were navigating the return to in-person school following extended remote learning in eight counties in Maryland, US. One element of the study was a survey implemented on the electronic data capture system, REDCap [4]. Other study elements included a household mailed survey and focus groups. The study was approved by the Johns Hopkins Medicine Institutional Review Board (IRB00290237) and participants provided informed consent.

To be eligible, survey respondents had to be a parent or guardian of a public-school student in grades K-8 during the 2021–2022 school year, live in one of eight counties in Maryland, and read English or Spanish. The REDCap survey was fielded from December 2021 to June 2022.

Participants were recruited through social media ads on Facebook, community flyers and listserv postings, and postcard advertisements sent to households sampled to reach underserved and rural families. The Facebook recruitment strategy was developed based on strategies described by Ali et al. (2020), who recommended the use of a “single image” advertisement format designed to send social media users to the survey link [3]. We used Meta’s Advertising Suite to direct advertisements to users who Meta categorized as parents of children between 6 and 17 years of age residing within our target counties. Recruitment materials referenced a $25 incentive for survey completion. Participants could choose to receive an electronic gift card by email or a physical gift card by mail and they provided relevant contact information to facilitate the remuneration they requested. Contact information for renumeration was not linked to survey responses to maintain respondent confidentiality.

Prospective participants followed a link to a public eligibility screener in REDCap. The eligibility screener was initially conceived simply to ensure respondents met eligibility criteria. The screener asked prospective respondents to indicate which of the eight target counties they lived in, confirm their status as a parent of a child in grades K-8 in a public school, and provide their zip code and how they heard about the survey so that recruitment could be monitored. Two initial survey security strategies were employed based on best practices for online survey security as outlined by previous work [13]. First, a basic CAPTCHA was included in the screener to prevent access by fraudsters using automated software to complete survey responses *en masse* for incentives. Second, a REDCap feature that uses browser cookies to prevent duplicate responses was activated. Those eligible on the screener then received a personalized link to the survey via email. Survey responses were carefully reviewed for internal consistency and data quality by two study staff members. Below we further detail the results of the initial fielding and iterative changes to the screener and survey.

## Results

**Fig 1:**
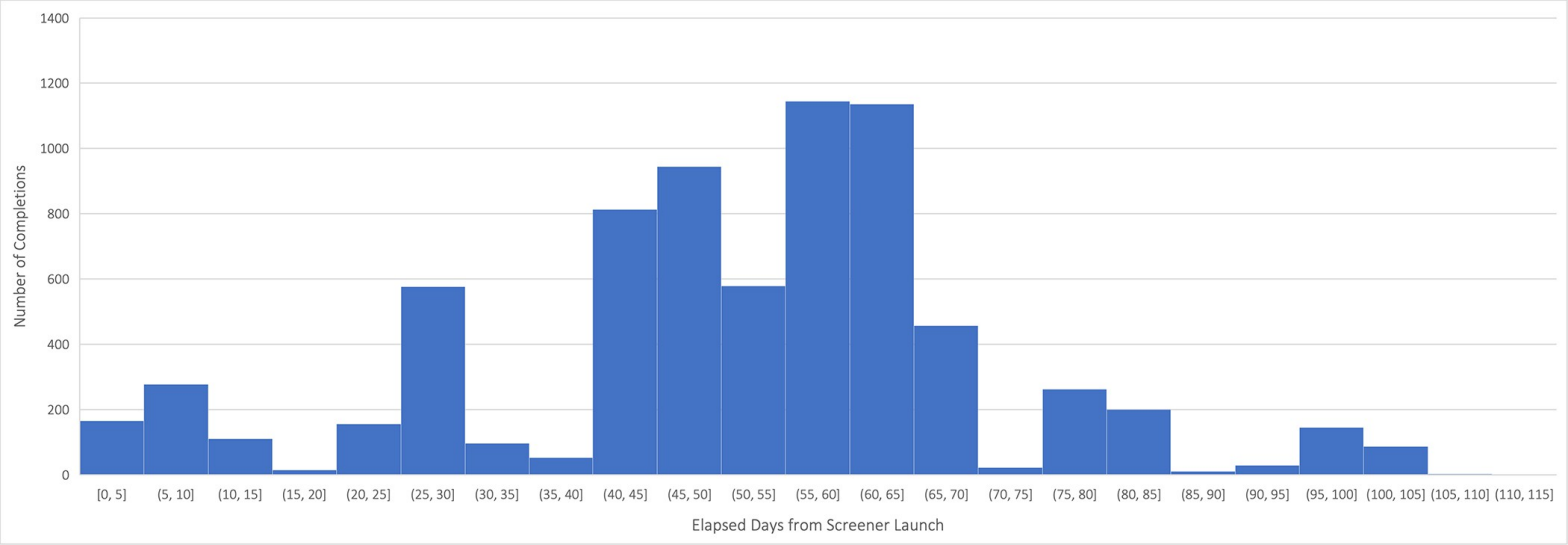
Temporal distribution of responses to revised survey eligibility screener following survey launch.

The screener and survey were initially fielded on December 22, 2021, at 5:30 PM EST, and disseminated via targeted Facebook advertising. **Fig 1** depicts the temporal distribution of screener responses throughout the survey period. After the Facebook advertisements had been running for one hour—with a reach of only approximately 125 individuals—2,578 screeners were attempted and 950 responses to the survey were completed, a volume highly suggestive of fraud. Among other suspicious indicators, responses came in batches of dozens per minute, overnight, and contained email addresses with strings of nonsensical letters and numbers. Whereas the advertisements were still classified by Facebook as “Pending Review” before 7:42 PM EST (i.e., the advertisement was not being delivered to members of the public), 260 responses appeared to have been submitted before the campaign received approval and began delivery. This suggests that fraudsters may have located the advertisement prior to when public delivery began; it remains unclear how this would have been accomplished.

When the suspicious activity was detected in the REDCap survey at 11:33 PM EST on December 22^nd^, the Facebook advertisement campaign was deactivated. Strikingly, between the opening and closing of the campaign, only two “link clicks” on the advertisement were logged—while over 2,500 screeners were attempted. This suggests that the majority of response attempts were not the product of organic engagement with the Facebook advertising campaign; rather, it is likely that these responses were the result of the dissemination of the survey link through other channels.

We hypothesized that an individual or group of individuals may have posted the public survey link to the eligibility screener to online communities that share information regarding the manipulation of incentivized research surveys—a phenomenon that has been previously documented [13]. It became clear that the screener was not adequately screening out fraudulent or suspicious respondents. As a result, the REDCap project was taken offline for redesign and Facebook advertisements were removed. Community-based recruitment and household mailings of recruitment postcards continued.

A revised REDCap eligibility screener that was harder to outwit was implemented in this new iteration of the survey fielding. For example, respondents were asked to choose their county of residence from a list of all counties in the state and to provide their zip code. Internal validation ensured that the participant lived in one of eight eligible counties and that their zip code was associated with an eligible county. Additionally, the respondent’s county of residence and zip code were required to be consistent between the screener questionnaire and the main survey if they were ultimately deemed to be eligible and received a survey link. A more complex CAPTCHA was added, as were two “speed bump” questions. Speed bump questions are designed to screen out bots and careless/automatic responders (an example of a speed bump question is, “The man couldn’t lift his son because he was so weak. Who was weak, the man or his son?”). Other types of speed bump questions include instructions for selecting an answer choice in the question stem that can be difficult to answer by automated scripts or rapidly responding fraudsters [14]. We also added a “honeypot question,” a question only visible to “bots” or other software tools that may be used for auto-responding [15]. However, we discovered that though we implemented this recommended strategy, it did not appear to be effective in screening out fraudsters—as no survey completions logged responses to this item.

On day 54 of survey fielding, to restrict access among respondents outside of the target area, a time zone difference calculation was implemented into the screener. This calculation compared the values of REDCap action tags “NOW” (i.e., the time reported by the client’s browser) and “NOW-SERVER” (i.e., the time reported by the institution’s REDCap server) to automatically calculate whether a respondent’s browser was set to a different time zone from the Eastern Time Zone—the time zone associated with the eligible study population. This calculation compared the client-side timestamp for screener completion with the server timestamp for receipt of responses. Respondents with greater than one hour difference were hypothesized to be attempting to complete the survey from alternate time zones and were thus barred from receiving the link for the study survey. Nearly one-quarter of responses for which time information was collected contained timestamp disparities. **Table 1** presents further data regarding timestamped survey responses. Notably, however, we detected fraudulent respondents who avoided detection by intentionally setting their client-side time to match the time zone associated with our study (Eastern time), suggesting that at least some fraudsters may be aware of time zone disparities as a potential flag for suspicious activity.

**Table 1:** Survey responses with time zone disparities.

Indicator	n
Responses containing timestamp information	4103
Responses with disparities between client and server	978
Responses with client-server disparities > 60 minutes	520

The revised survey eligibility screener was deployed on January 26^th^, 2022. However, it became clear that our additional security enhancements did not sufficiently reduce the number of fraudulent attempts to complete the screener and many survey responses continued to be flagged as suspicious. Such flagged responses required additional review (for example, we triangulated responses to duplicate questions between the screener and survey to ensure internal consistency). Out of a total of 9,760 REDCap records, 978 had evidence of time zone disparities, and 316 respondents marked two or more counties in the screener when asked for their primary county of residence.

To help screen potential responses for signs of fraud more efficiently, we implemented automated strategies deployed within REDCap to prevent the completion and submission of responses demonstrating clear signs of fraud. In addition, we developed a points-based methodology for manually identifying potential signs of fraud, derived from prior published guidelines [1, 2, 10]. This method involved detailed review and cleaning by study staff. Deriving guidance from the work of Ballard et al. (2019) we assigned points to observed indicators of suspicious activity, with point values corresponding to the degree that an indicator suggested fraud. **Table 2** provides an overview of our points-based methodology. Two or more points would cause a response to be marked as fraudulent. This system was developed based on pre-testing data and using responses we knew were valid. Prior to implementation, the system was tested to ensure that past “known-good” responses would not be flagged as fraudulent.

**Table 2:** Points assigned to various indicators of possible fraud in study algorithm.

Point Value	Indicators of Possible Fraud
1pt	Completion time infeasibly short (<8 min) • Pre-testing found valid completion times from 8-20 min (median 17 min)
1pt	Same county + zip code as responses submitted to REDCap simultaneously
1pt	Grouping of 3+ responses submitted to REDCap with identical/similar responses
1pt	Unusual email format (e.g., asfs241421@email.com) • Alternatively, misspellings or extraneous letters/numbers (e.g., hannaahsmiithweka3242@email.com)
1 pt	Incorrect speed bump questions
1pt	Reported inactive or nonexistent recruitment source • Selecting “Facebook Ad” when advertisements were inactive • Selecting “Radio Advertisement” when the format was never used
1pt	Duplicate IP address
1pt	Submission to REDCap in waves/at regular intervals (e.g., 3 responses every 5 min) • We considered batches of responses submitted within 60 seconds at regular intervals
2pts	Inconsistency in response/conflicting information within or between survey forms • Inconsistency in data reported between eligibility screener and survey • Inconsistency between duplicate questions in the same form • Child age inconsistent with their reported grade (e.g., 5 years old in 8^th^ grade) • Reporting incorrect or non-existent zip codes
2pts	Response to optional open-ended question text identical to another respondent
2pts	Time zone difference from Maryland (EST)
2pts	Email/address/telephone number already reported by another respondent

On day 75 of survey fielding, our institution’s instance of REDCap launched a form of anonymized Internet Protocol Address (IP) logging, which we immediately incorporated into our project. Prior to this, due to privacy concerns, our instance of REDCap did not provide any IP logging or geolocation features. This newly-added feature anonymized the respondent’s IP address (IP addresses are considered personal health information (PHI) under the Health Insurance Portability and Accountability Act (HIPAA)) but allowed us to identify multiple respondents from the same IP address. This additional feature allowed us to identify those who completed the survey more than once from the same IP. This feature was not used as a rejection criterion but was rather utilized during the review of survey responses for data quality because two legitimate respondents from different households can share one IP address. It should be noted that this form of anonymized IP address logging does not permit the identification of fraudsters who use multiple IP addresses, such as via Virtual Private Servers (VPS), Virtual Private Networks (VPN), proxies, spoofed IP addresses, or methods of obfuscation [1]. We considered duplicate IP addresses to be one point in our algorithm for quantifying the likelihood of fraudulent activity (**Table 2**). **Table 3** includes a timeline of the survey security steps implemented across the survey fielding period.

**Table 3:** Timeline of survey security strategy implementation.

Elapsed days from survey launch	Strategy
1	Launched revised REDCap eligibility screener, including CAPTCHA and speed bump questions
54	Implemented time zone difference calculation into eligibility screener
75	Implemented IP logging feature
126	Created siloed copy of REDCap survey

IP: internet protocol

### Further observations

On day 126 of fielding, we created a copy of the REDCap project, with the same settings as the existing project, which was used specifically for Facebook recruitment directed toward Spanish-speaking respondents. This copied project was accessible to respondents via a different weblink. This copy presented numerous benefits for data integrity. We hypothesized the weblink to our original survey had been shared within covert networks of fraudsters [13]. The use of surveys accessible through different weblinks may present an opportunity for researchers to limit the efficacy of such networks.

## Discussion

In fielding a survey of parents designed to capture perceptions about the safe return to school during the COVID-19 pandemic, we encountered a variety of threats to data integrity that required substantial time and effort to identify and address. The study team included a programmer analyst, as well as an individual with experience in social media campaigns, and received consultation from the REDCap administrator for our institution. The expertise of these individuals was particularly useful in our response to these threats.

Mechanisms to prevent fraud should be an integral consideration during the survey design phase. REDCap administrators should be aware of these mechanisms and recommend that all surveys implement measures to maintain data integrity and combat fraud. However, it is important to recognize that even existing recommendations can be circumvented by fraudsters with adequate resources. Automated measures to detect indicators of fraudulent activity and bar respondents demonstrating suspicious behavior from completing the survey can be “brute-forced” by fraudsters, who may attempt thousands of responses to determine a pattern of response that allows them to be deemed eligible. Upon determining such patterns, fraudsters will repeatedly utilize these strategies until they are detected.

To limit the disruptions associated with needing to close surveys that have been compromised, here we outline several strategies. Best-practice recommendations for other researchers, based on our findings, are summarized in **Table 4**.

**Table 4:** Summary of recommendations and best practices for reducing likelihood of web-based survey fraud.

Recommendations
1. Make duplicate/siloed survey instances available through different links to minimize link sharing
2. Remove terms “survey” or “study” from the text of the survey weblink (for example, “redcap.link/example” rather than “redcap.link/examplesurvey”)
3. Activate IP logging features, if available
4. Enable time zone checks to ensure respondent’s computer is in the target time zone, if applicable
5. Add speed bump questions to promote respondent attentiveness and reduce automatic responses
6. Include false/impossible response options (such as a selection for “TV ad” when recruitment never occurred through TV ads) to flag suspicious responses
7. Use within-survey validation (e.g., ask county and ZIP code of residence at multiple points to ensure consistency in responses, or ask the identical question more than once)
8. Deploy cross-survey form validation (e.g., ask the same question on an eligibility screener and survey to compare responses for consistency across forms)
9. Include an open-ended response survey question to flag nonsensical or irrelevant content

### Strategies deployed within the data collection platform

Some strategies can be deployed directly through data capture or survey implementation platforms. First, consider omitting words like “survey,” “study,” or “research” from the text of survey platform weblinks to make them harder for fraudsters to find. Also, if possible, create multiple surveys associated with different web addresses. Cloned copies of the REDCap project allow researchers to rapidly change links as needed. Should the link for one survey instance be compromised by malicious actors, researchers can quickly pause the affected instance while permitting alternative instances to continue normal function. Pausing an instance to reduce fraud risk could impact the composition of the survey sample. However, the impact of these pauses depends on factors including frequency and duration of the pause and sampling strategy and study design. For example, studies with complex sampling designs are likely to be more impacted by paused survey links than those that rely on convenience samples. More work is needed to fully understand the potential impacts of frequent or extended pauses of siloed survey instances.

If available, researchers should activate IP logging, either direct IP logging or a HIPAA-compliant anonymized alternative that allows multiple responses from the same IP address to be flagged. (Note that even legitimate responses may contain duplicate IP addresses, as respondents may live or work where one IP address is associated with multiple households). If survey participants are restricted geographically, implement automatic comparison of client-side and server-side time stamps to identify responses completed outside of the target geography. However, it is important to know that fraudsters may adjust their computer clocks to circumvent these calculations.

### Strategies in the survey design phase

Researchers should consider a two-stage process: an eligibility screener with a subsequent personalized survey link by email for those who are deemed eligible on the screener. Several design features can make it easier to reject or identify suspicious responses. In the screener, consider including a question with a false or impossible answer choice that could be used as a flag for suspicious activity. For example, a question about where participants heard about the survey could include “television ad” when no television ads were used. Add questions that are difficult for bots or computer algorithms to answer. For example, “speed bump” questions can help ensure respondents are reading the questions rather than answering indiscriminately.

An important way to identify suspicious activity is to validate the information respondents provide, within and/or across survey forms, depending on the study design. Validations within the same survey or screener can also be used to thwart “brute force” efforts to determine study eligibility criteria. For example, we asked participants to choose their county of residence from a list (including counties that were not eligible) and their associated zip code. Counties and zip codes were automatically compared and responses that did not match were deemed ineligible. Validations can also be used between two survey forms. For example, we asked participants to provide their county and zip code on both the screener and again on the emailed personalized survey. If the information did not match, responses were flagged as suspicious. We also included the same question more than once on the personalized survey as a data quality/internal consistency check. While participants’ answers may vary for reasons unrelated to fraud, consistency is an important data quality indicator.

Finally, consider adding an open-ended response to survey forms such as “Is there anything else you’d like to share with us on this topic?” Open-ended responses can be reviewed for nonsense or unrelated content. Multiple survey responses that include the same or similar text can also be used to identify responses completed automatically.

It is important to note that even if these suggested strategies are used, human monitoring and oversight are critical. Human oversight is needed to monitor responses for new or emerging threats to data integrity and to review activity flagged as suspicious to avoid inadvertently excluding eligible participants with valid responses. To avoid wasting research funds and further incentivizing fraudsters, survey remuneration should never be automated and should always include a human review. Other strategies such as confirming the identity of a participant with an email or phone call before releasing funds may also help but raise questions about ethical obligations to remunerate participants who are non-responsive to researchers’ efforts to contact them.

## Conclusions

Internet-based research has become increasingly common because it is comparatively inexpensive, less labor intensive than in-person data collection, and may expand access to a wider pool of potential research participants. Nonetheless, researchers must recognize threats to survey integrity posed by fraudsters who aim to gather study payments. Because fraudsters are constantly updating their approaches, continuous, ongoing monitoring is needed. Using strategies like screeners with embedded data quality questions and validation, time zone restrictions for completion, IP address logging, and multiple data forms can reduce threats to research integrity.
